# Complex Genetic Architecture of Cardiac Disease in a Wild Type Inbred Strain of *Drosophila melanogaster*

**DOI:** 10.1371/journal.pone.0062909

**Published:** 2013-04-29

**Authors:** Zhi Zhang, Benjamin Hsieh, Amy Poe, Julie Anderson, Karen Ocorr, Greg Gibson, Rolf Bodmer

**Affiliations:** 1 Development and Aging Program, Neuroscience, Aging and Stem Cell Research Center, Sanford-Burnham Medical Research Institute, La Jolla, California, United States of America; 2 School of Biology, Georgia Institute of Technology, Atlanta, Georgia, United States of America; Heart Science Centre, Imperial College London, United Kingdom

## Abstract

Natural populations of the fruit fly, *Drosophila melanogaster*, segregate genetic variation that leads to cardiac disease phenotypes. One nearly isogenic line from a North Carolina peach orchard, WE70, is shown to harbor two genetically distinct heart phenotypes: elevated incidence of arrhythmias, and a dramatically constricted heart diameter in both diastole and systole, with resemblance to restrictive cardiomyopathy in humans. Assuming the source to be rare variants of large effect, we performed Bulked Segregant Analysis using genomic DNA hybridization to Affymetrix chips to detect single feature polymorphisms, but found that the mutant phenotypes are more likely to have a polygenic basis. Further mapping efforts revealed a complex architecture wherein the constricted cardiomyopathy phenotype was observed in individual whole chromosome substitution lines, implying that variants on both major autosomes are sufficient to produce the phenotype. A panel of 170 Recombinant Inbred Lines (RIL) was generated, and a small subset of mutant lines selected, but these each complemented both whole chromosome substitutions, implying a non-additive (epistatic) contribution to the “disease” phenotype. Low coverage whole genome sequencing was also used to attempt to map chromosomal regions contributing to both the cardiomyopathy and arrhythmia, but a polygenic architecture had to be again inferred to be most likely. These results show that an apparently simple rare phenotype can have a complex genetic basis that would be refractory to mapping by deep sequencing in pedigrees. We present this as a cautionary tale regarding assumptions related to attempts to map new disease mutations on the assumption that probands carry a single causal mutation.

## Introduction

One of the major unresolved issues in human genetics concerns the genetic architecture of rare non-Mendelians disorders that have high heritability [1]. Psychological conditions such as autism, intellectual disability, and schizophrenia; craniofacial abnormalities; acute respiratory disorders; and the spectra of cardiac arrhythmias collectively affect several percent of the population but are only partially explained by existing genetic models. It is assumed that rare variants of large effect contribute [2], and recent whole exome sequencing studies confirm their involvement in at least a minority of cases of some of the above diseases [3]–[9], but they are more likely necessary than sufficient. Likewise, the contribution of rare variants to complex diseases, including heart disease, is yet to be clarified [10].

Cardiovascular disease is the leading cause of death in the United States and is a major cause of disability [11]. Susceptibility is modified by both environmental risk factors and genetic factors, and mutations in genes involving numerous biological and metabolic pathways have been shown to modify the risk for heart disease [12]. Heritability estimates for heart disease from family and twins studies range from 17% to 61% of the observed variation in in the study populations [13], [14]. Genome-wide association studies have localized over 30 loci that contribute to coronary artery disease [15], [16], and numerous associations with cardiac arrhythmia have also been reported [17], yet in both cases less than 15% of the variation for liability has been explained.

The generic explanation for high heritability is now the infinitesimal model, namely that hundreds if not thousands of variants with a range of effect sizes and allele frequencies individually explain a small fraction of genetic risk in the population [1], [2], [18]. The problem with this model for rare disorders is that it does not readily explain the high sibling recurrence in a small numbers of families [19], [20]. Instead, theory points to multiplicative interactions between several rare variants. A few hundred rare variants, each with genotype relative risks in the range of 2 to 5, and at frequencies around 1 percent, can lead to familial clustering of disease [21], but genome-wide association studies are not powered or designed to detect such variants. It is also questionable whether whole genome sequencing will typically provide more clarity with respect to this conundrum. It is possible that experimental organisms can help, since they offer the resolution of controlled genetic mapping.

*Drosophila* is well-known as a model for studying the mechanisms by which human disease genes cause pathology [22], [23], including heart disease [24]–[28], but it is less well appreciated that they may also model the genetic architecture of disease, since flies presumably also have diseases that have a genetic basis. For example, in a quantitative evaluation of 50 highly inbred lines of wild type *D. melanogaster*, we provided evidence that at least nine lines exhibited age-dependent heart rhythmicity [26]. Six lines actually showed resistance to pacing-induced heart failure at old age, while three others were already highly susceptible and arrhythmic at a young age, and a major-effect locus was mapped to the tip of chromosome 3R in the WE01 line [26]. We reasoned that most of these aberrant inbred line effects would be due to capture of rare variants of large effect as homozygotes, allowing the variants to be mapped rapidly using contemporary genomic approaches. This report shows however that at least for this one case, WE70, the simple model for monogenic disease causality seems to be incorrect and that a highly penetrant rare condition has a very complex genetic basis.

## Results

### Phenotypic Analysis of Arrhythmia and Cardiomyopathy in WE70 Flies

**Figure 1:**
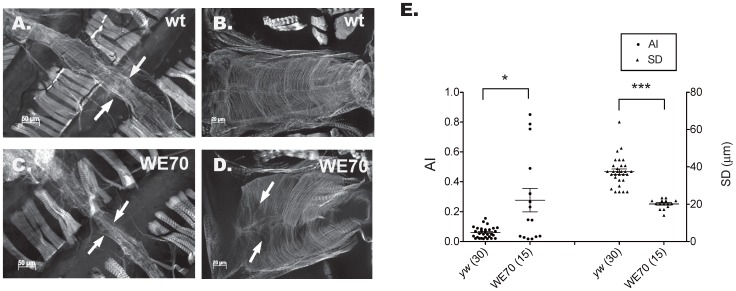
Cardiomyopathy and Arrhythmia in WE70. (A-D) Confocal z-stack of 1-week old adult hearts for laboratory wildtype (*yw*) and WE70 strains stained with phalloidin at 10X and 20X magnification (B,D: conical chamber). (A,B) *yw* heart shows normal cardiac tube diameter (arrows in A) and regular myofibrillar organization. (C,D) WE70 heart shows narrow heart tube (arrows in C), and myofibrillar disorganization (arrows indicate gaps in D). (E) Heart function parameters of 1-week old adult hearts for *yw* and WE70 strains (AI: arrhythmia index; SD: systolic diameter) [24], [25]. WE70 shows both increased AI and increased variance of AI among flies, compared with *yw* wildtype flies. SD of the heart tube in 1-week old WE70 females is narrower than in 1-week old *yw* females (similarly, the diastolic diameter is also reduced; all parameters listed in Table S1). Data are analyzed by t-test, *p<0.05, **p<0.01, ***p<0.001, otherwise p>0.05 not indicated.

Video imaging of the adult heart [25], [29] in WE70 flies revealed two phenotypes resembling human cardiac arrhythmia and restrictive-like cardiomyopathy [26]. Moreover, phalloidin staining of paraformaldehyde-fixed dissected hearts [24], [29], [30] indicated disorganization of the myofibrils in the conical chamber relative to laboratory wild-type (*yw*) controls (Fig. 1A–D). Cardiomyopathy is evident at one week of age as reduced fractional shortening (see Table S1), in particular a significant reduction in the systolic cardiac diameter (SD), from 37±1(SEM) µm in normal adults to 20±0.7 µm in WE70 (Fig. 1A, C, E). Arrhythmia is defined by an index of variance of the beat length normalized to the median heart period of each fly (Arrhythmia Index, AI), measured over a 30 second time interval in 15–30 flies [25], [29], [31]. In WE70 AI has a mean value of 0.28, which is significantly greater than the mean AI of 0.06 observed in wildtype (Fig. 1E) (Table S1, Video S1, S2).

**Figure 2:**
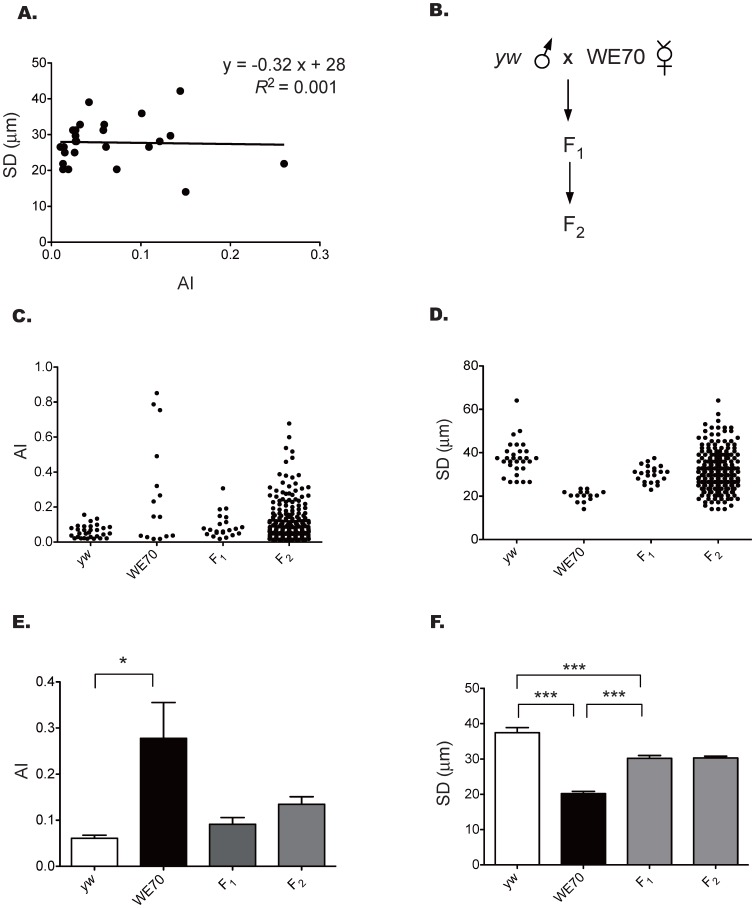
Phenotypic segregation in the WE70 × *yw* cross. (A) No significant correlation overall between the arrhythmia index (AI) and the systolic diameter (SD) phenotypes in F_2_ progeny is observed (R^2^ = 0.001, F-test, p = 0.88). (B) Crossing scheme design between *yw* and WE70. (C, D) Distribution plots of AI and SD by genotype showing incomplete penetrance of elevated AI in WE70 and the dispersion of phenotypes in the F_2_ with approximately one-quarter aberrant flies. (E, F) Mean of AI and SD among WE70, *yw*, F_1_ and F_2_. The error bar is representing S.E.M. of each group. The significant differences in AI and SD between WE70, *yw*, and F2 are indicated (t-test, *p<0.05, **p<0.01, ***p<0.001).The F_1_ phenotype is intermediate between *yw* and WE70 for both AI and SD, but generally closer to *yw*.

It is possible that disrupted contractility (reduced SD) in WE70 is due to elevated arrhythmia, or vice versa. This does not seem to be the case, since there is no correlation between systolic diameter (SD) and arrhythmia index (AI) (R^2^ = 0.001, F-test p = 0.88 measured on 25 adult female F_2_ progeny from a cross of WE70 to the standard *yw*-Oregon R lab stock) (Fig. 2A). This implies that there are at least two distinct genetic risk factors (mutations or polygenic polymorphisms) promoting heart disease in the WE70 strain.

In order to test whether AI and SD follow monogenic or polygenic inheritance, we examined the phenotypes in a classical Mendelian F_1_–F_2_ crossing scheme of WE70 crossed to the laboratory wildtype *yw* strain. The F_1_ progeny show an intermediate phenotype that resembles *yw* more than WE70, while approximately one quarter of the F_2_ progeny (sibling crosses of F_1_) show WE70-like SD and AI phenotypes (Fig. 2C–F), consistent with a recessive model of monogenic disease. However, we note that the arrhythmia is incompletely penetrant (a minority of the WE70 stock have regular heart beats), and that there is a continuous gradation of the systolic diameter measure in the F_2_ despite relatively clean phenotypic separation of the two parental strains (Fig 2C–F), perhaps more suggestive of an oligogenic architecture.

### Initial QTL Mapping of Arrhythmia and Cardiomyopathy in WE70 Flies

**Figure 3:**
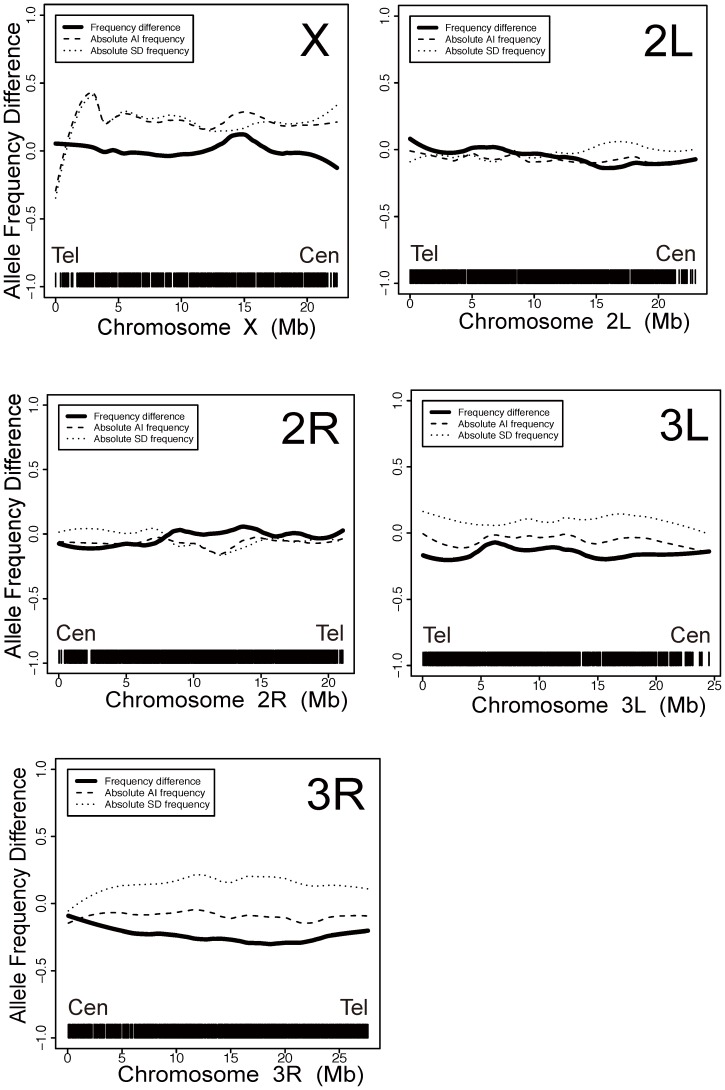
Bulked Segregant Analysis (BSA) by Single Feature Polymorphisms (SFP). Each panel shows the sliding window average of SFP intensity for greater representation of the WE70 allele (positive) or *yw* allele (negative) in arrhythmic or narrow diameter flies. The entire X chromosome has the expected enrichment for WE70 alleles inherited from the F_1_ fathers. Shallow peaks are observed for SD near 15MB on 2L, at both the tip and centromere-proximal third of 3L, and along most of the length of 3R. For AI there is a minor peak at 15MB on the X. Both traits show a shallow protective effect between MB 15 and 20 on 2R. There is no evidence for a major QTL for either trait (Cen : centromere; Tel : telomere).

In order to map the genetic variants for AI and SD in WE70 flies, we used single feature polymorphism (SFP) analysis [32], [33] to contrast the genome-wide genotype frequencies between pools of flies with aberrant rhythmicity or systolic diameter resulting from F_2_ progeny of the cross of *yw* to WE70. SFP analysis is an indirect method for genome-wide genotyping that utilizes differential hybridization of genomic DNA to probes on a DNA chip that was initially designed for gene expression profiling, but can be used for species where genotyping chips are not available. DNA was prepared from three independent pools of 15 flies for each of the two types, as well as from the two parental lines. The samples were sheared and labeled with biotin, then hybridized to Affymetrix *Drosophila* expression microarray chips. Mismatch hybridization, namely a significant difference in the hybridization intensity between the parental lines, was detected at approximately 4% of all perfect match (PM) probes, located in over 9,000 probes with an estimated False Discovery Rate of 11% (see Materials and Methods). This is consistent with the known levels of polymorphism in *Drosophila melanogaster* where typically 3% of nucleotides differ between any two chromosomes, a subset of which will affect short oligonucleotide hybridization. Figure 3 contrasts the average signal intensity for these mismatch probes between the triplicate SFP signals of the high AI and low SD flies (dashed lines) as well as the difference between the two pools (solid line). SFP signals are inherently noisy, so sliding windows of the average difference in hybridization intensity between the replicate normal and disease classes over 100 consecutive probe-sets were computed. Significance thresholds were assessed by permutation of mixed combinations of the SFP arrays as described in the Materials and Methods. The plots are analogous to QTL profiles, where peaks or troughs in the distribution represent enrichment for WE70 or *yw*-derived genotypes respectively.

It is immediately evident from these profiles (Fig. 3, raw SFP data see Table S2) that both phenotypes are genetically complex. There is not a single peak for either phenotype where all DNA of the affected flies is derived from WE70, indicating that the disease phenotype is not likely due to a single mutation with high penetrance. Both pools show an excess of WE70-derived signal on the X-chromosome due to the fact that all F_1_ males carry complete WE70 X-chromosomes, confirming that the SFP strategy does detect expected genetic differentiation. There is just a single suggestive peak for arrhythmia toward the base of the X chromosome, while the centromere-proximal half of 2R tends to be derived from *yw*. For SD, there is an excess of signal along the full extent of 3R, suggesting multiple genetic factors. Furthermore, troughs in the distribution for both traits on 2R are consistent with the *yw* strain also carrying alleles that contribute at least to the arrhythmia phenotype. In most cases, peaks and troughs that exceed the permutation threshold were also observed in the profiles of individual pools (data not shown), indicating that there is both technical and biological repeatability to the analysis. However, the resolution is too low to support mapping to individual loci. Previous studies of Arabidopsis using essentially the same strategy and pool sizes led to the localization of a single mutation affecting the phenotype [33], and the experiment reported here should have been powered to detect such a factor. We conclude that multiple loci contribute to both aspects of the cardiac disease phenotype in WE70.

### Chromosome Substitution and Recombinant Inbred Lines (RILs) Reproduce the WE70 Phenotypes

**Figure 4:**
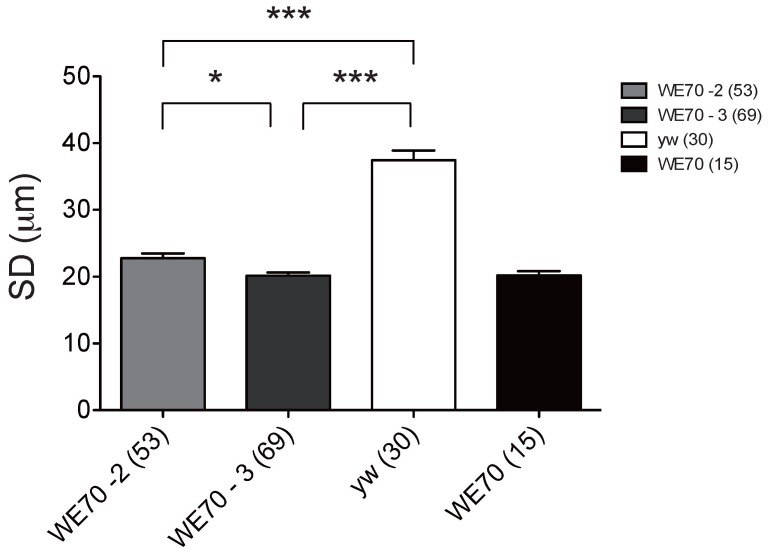
Chromosome substitution regenerates the dilated cardiomyopathy phenotype. SD for wildtype *yw* is ∼38 µm. Either chromosome substitution alone (WE70-2, WE70-3) also has an average systolic diameter of ∼20 µm, which is not significantly different from the WE70 phenotype. The number of hearts examined is indicated in parenthesis, and whiskers show one standard deviation unit. Significant pair-wise comparisons are indicated (*p<0.05, **p<0.01, ***p<0.001, otherwise p>0.05 not indicated).

**Figure 5:**
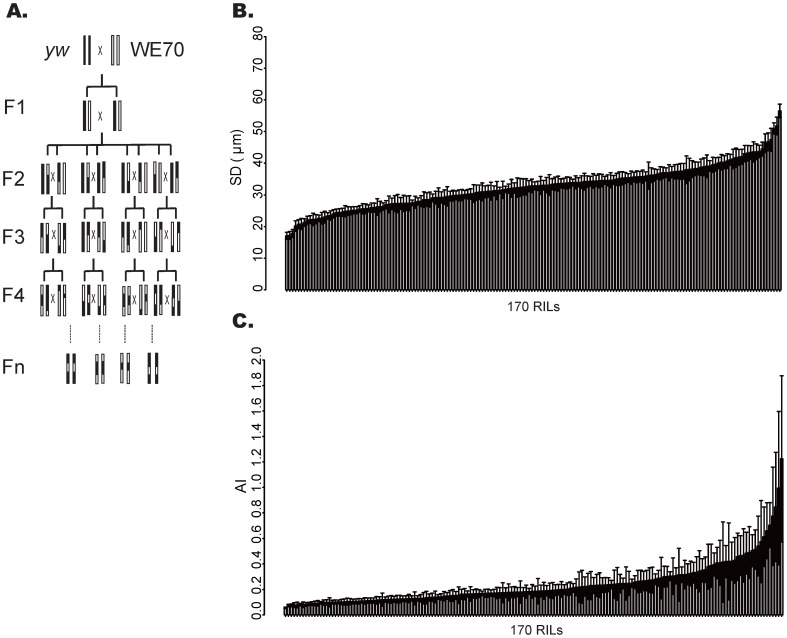
Distribution of phenotypes in RIL. (A) Crossing scheme employed to generate a panel of 170 RIL containing fragments of the *yw* and WE70 genomes. (B,C) RIL SD and AI are ranked from smallest to biggest. Six RILs with most extreme phenotypes, smallest and biggest values of both SD and AI, were taken forward for whole genome sequencing.

Nevertheless, the highly penetrant nature of the phenotype leads us to suspect that the risk factors are likely to be isolatable in recombinant inbred lines that would also provide finer resolution mapping than SFP analysis. As a prelude to this effort, we generated chromosome substitution lines, where balancer chromosomes were used to cross each of the two major WE70 autosomes into the *yw* background independently. Figure 4 shows the surprising result that either the second or the third WE70 chromosome alone in homozygous form is sufficient to regenerate the cardiomyopathy phenotype. There is a slightly greater impact of the third chromosome, but neither substitution is statistically different from the pure WE70 line. This confirms the polygenic architecture of the trait, and also implies that the disease phenotype is non-additive. That is to say, summation of effects across the genome does not lead to incremental worsening of the phenotype. In order to assess whether the effect could be isolated to specific chromosomal regions, we generated a panel of 170 recombinant inbred lines (RILs) by 12 generations of pair-mating of flies derived from the hybrid cross of *yw* to WE70 (Fig. 5A). This level of inbreeding is expected to result in >90% homozygosity in genomic regions that do not carry recessive lethals. Measurement of the two phenotypes in 10 dissected adult hearts of each RIL results in the phenotype distributions shown in Fig. 5B,C (see also Table S3). The majority of the RILs have somewhat elevated AIs (mean 0.24, range 0.06 to 1.22) and intermediate SDs (mean 33 µm, range 17–57 µm) considered in the wildtype range, but several lines clearly show extreme disease phenotype (low SD or high AI) with high penetrance, while a few others have an opposite extreme phenotype, ie. very large SD and AI. Over 90% of the variance for SD and 33% for AI was between different RILs (as opposed to between individual flies within each RIL), confirming a strong genetic component for both traits. The Pearson correlation between AI and SD was non-significant (R = 0.07, t = 0.86, p = 0.39), again confirming the genetic independence of the traits (Fig. S1), as does the separation of the lines with ‘disease classification’ into different RILs. In contrast, when we compare the diastolic diameters (DD) with the SD of the RILs, we find a strong correlation (R = 0.94, t = 35.21, p<0.0001) (Fig. S2, S3).

**Figure 6:**
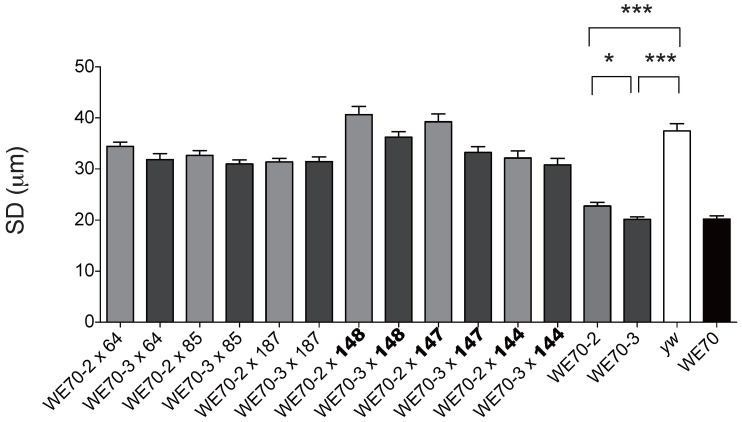
Epistatic interactions may account for the systolic diameter phenotype. RIL 64, 85 and 187 (see Table S3) each have an extreme narrow diameter similar to the parent WE70 line, whereas RIL 147, 148 and 144 (indicated by bold) have wildtype-like diameters. Each was crossed to either the second (WE70-2, light gray) or third (WE70-3, dark gray) chromosome WE70 substitution lines. Progeny from all the crosses show intermediate to wildtype heart diameters. Significant pair-wise comparisons are indicated (*p<0.05, **p<0.01, ***p<0.001). All other comparisons relative to *yw* (white bar) were not significant. WE70 parental line, black bar on the right, is also shown for comparison. 12–30 flies of each line were analyzed.

In an attempt to confirm that the extreme RILs for systolic diameter harbor discrete loci whose effects can be observed in defined crosses, we next performed complementation testing between extreme RIL and the 2^nd^ and 3^rd^ chromosome substitution lines (WE70-2 and WE70-3, respectively). We expected that the RILs with extreme heart constriction phenotypes to fail to complement one or both of the two chromosome substitution lines. However, unambiguous complementation of the disease phenotype was observed in each case, only mildly reducing the diameter in some cases, compared to wildtype *yw* (Fig. 6). In particular, the progeny of RIL 64, 85 and 187 with extreme narrow SD crossed to WE70-2 and WE70-3 did not show the aberrant constricted ‘disease’ phenotype, implying that there are multiple factors in each chromosome that must combine together to reproduce the parental cardiomyopathy phenotype. Although each RIL should on average carry as much WE70 DNA as either chromosome substitution line, and hence each hybrid F_1_ fly should have as many homozygous WE70 regions across both autosomes (ie. compared to homozygous WE70-2 or WE70-3 lines), these combinations are not sufficient to regenerate the parental WE70 phenotype in a significant manner. This again is consistent with non-additivity of the genetic effects, and also suggests a significant degree of phenotypic epistasis.

### Whole Genome Sequencing Fails to Resolve Independent Disease Loci

**Figure 7:**
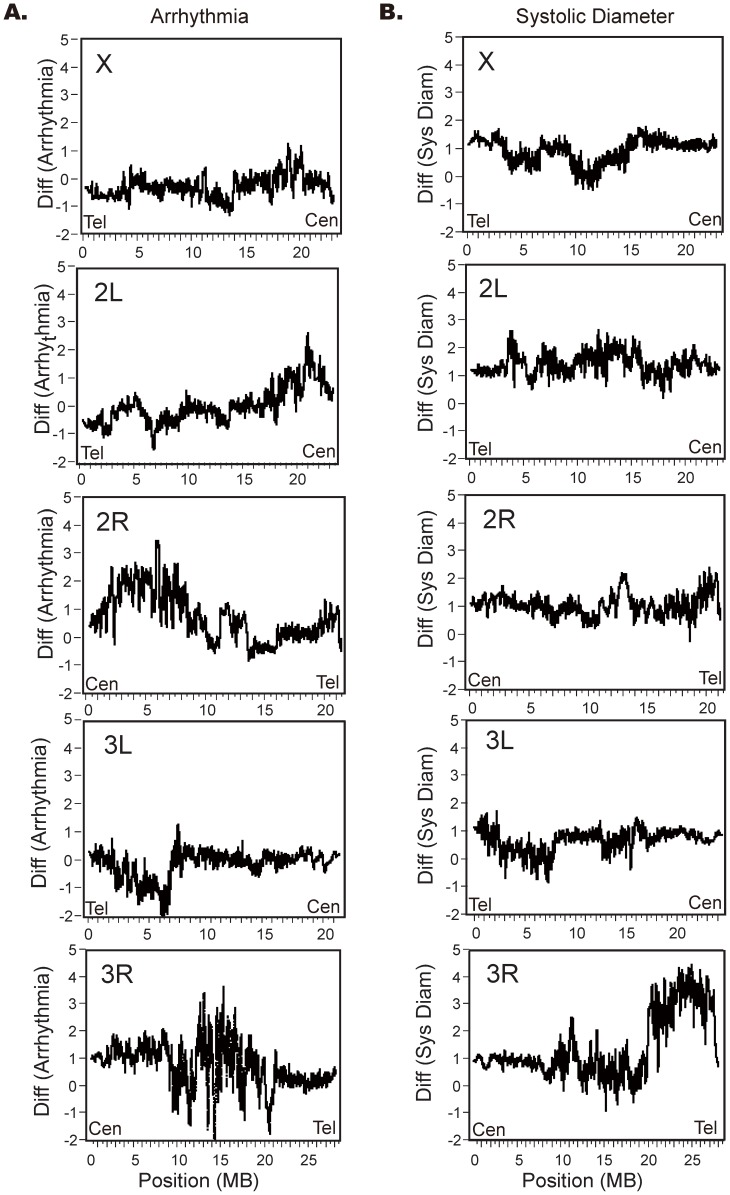
QTL profiles from re-sequencing of RIL. For each phenotype, we computed the average number of WE70 or *yw* alleles in each of 6 RILs with extreme phenotypes, based on a high confidence set of 100,000 SNPs distributed at approximately 10 kb intervals. The profiles represent the ratio of the two allele types in sliding windows of 100 SNPs (Cen : centromere; Tel : telomere).

Finally, in an attempt to map which regions of the RILs may harbor major effect loci, low pass (∼8X average coverage) whole genome sequencing of 23 RILs representing 5 or 6 lines with each of the four extreme phenotypes (smallest or biggest SD or AI; Fig. 5B,C; Table S3) was performed. Residual heterozygosity in the lines was sufficiently high, likely owing both to incomplete inbreeding of the RIL and incomplete homozygosity of the WE70 parent, so breakpoints could not be mapped with certainty. Consequently, we again turned to a sliding window analysis to map the linkage of genotype with phenotype in the two pairwise contrasts of disease and normal. Figure 7 plots the ratio of parental alleles along the chromosome, with peaks representing departure from the null 50–50 mix of WE70 and *yw* contributions with permutation again used to establish significance. One strong peak was observed for each trait, encompassing the distal third of 3R for systolic diameter, and the proximal half of 2R for arrhythmia. Neither of these was present in all of the aberrant RIL, so the effect of any variant in each interval is not sufficient to produce the phenotype. As with the SFP analysis, there are multiple peaks beyond permutation-computed thresholds, but none of these are consistent with single-gene segregation in the affected lines. There is also only partial overlap between the location of RIL and SFP peaks, suggesting that neither technology has the power to resolve all of the genetic factors.

Annotation of the genome sequences reveals a small number of candidate mutations that are expected to be deleterious with respect to protein function. The SeqAnt tool was used to identify a complete list of non-synonymous coding variants and these were annotated as probably or possibly damaging on the basis of their predicted effect on protein structure and/or disruption of an evolutionarily conserved residue. Table S4 lists all of the stop and frameshift coding mutations in WE70, as well as a number of amino acid changes in proteins that are encoded by genes previously associated with heart phenotypes. Further studies would be required to demonstrate that any of these, or one of the myriad non-synonymous substitutions (not shown), contribute to the phenotype.

## Discussion

Each of the four experiments described above lead to the conclusion that an apparently simple rare disease phenotype in *Drosophila* actually has a complex genetic architecture. Multiple loci are implicated, and they probably interact in multiplicative and epistatic ways in order to explain the complementation patterns between the various derivative lines. We know that in *Drosophila* similar (heart) disease phenotypes to those in humans can be produced by mutagenesis and ascribed to single gene mutations [24], [25], [27], [34], [35], but our analysis of this naturally occurring case shows that a highly penetrant rare disease can also have a polygenic basis. This is sobering news with respect to efforts to define the causal basis of rare conditions.

In approaching these experiments, our hypothesis was that near-isogenic lines would efficiently capture rare variants of large effect. In fact, the distribution of disease phenotypes places an upper limit on the number and frequency of fully penetrant mutations. For example, if 50 disease-sufficient mutations were segregating at a frequency of 1%, then at least one third of all Nearly Isogenic Lines extracted from wild populations would be expected to be homozygous for one of them and the prevalence of disease would be approximately of that order. Since fewer than 10 percent of lines are arrhythmic or constricted, no more than 10 recessive heart disease variants at 1% frequency are likely to be segregating in wild populations, or no more than ∼100 at 0.1% frequencies [26]. It is possible that some of the other highly inbred lines that we initially screened [26] harbor such variants, but the analysis of WE70 shows that other genetic models must also be considered.

Given the existence of rare variants with genotype-relative risks that more than double the likelihood of disease for carriers, individuals are actually considerably more likely to be multiply heterozygous for two or more such rare variants than homozygous for one of them. For example, 200 variants at 1% frequency will generate a frequency distribution of the number of rare variants per individual centered around one or two, but with a tail of up to 8 variants in a sample of several thousand individuals. Under standard multiplicative models, individuals who have 5 or more of these variants have substantially elevated risk of disease: if the population prevalence is 1% and average individual genotype relative risks are 2-fold, then the joint likelihood of disease with 5 variants whose effects multiply together is over 50%. This can generate high heritability, and high sibling relative risks in families where multiple variants happen to be segregating [19]–[21]. Such a model could at least partially explain the complex architecture observed here, with the caveat that the chromosome substitution lines imply that not all interactions are equivalent in their likelihood of promoting disease.

Translating these fly data to the human situation suggests one explanation for the failure of whole exome sequencing approaches to identify causal variants for rare diseases such as autism and intellectual disability in more than a minority of cases [3]–[7]. The types of variant that contribute to multiplicative interactions need not be as damaging to the proteins as those documented in disease mutation databases, and they could be regulatory. There is little statistical theory to support the proposition that they will be identifiable from individual or even family-based sequencing. Considerably more fine-structure mapping than that described here could lead to the isolation of rare variant polygenes, but it will entail somewhat risky investment of time and money. Possibly a better strategy proving the worth of the *Drosophila* model will be to screen fully sequenced high quality genomes such as that of *Drosophila* for candidate mutations in genes known to be involved in specific organ functions, cross these lines together and then use genotypic selection to breed lines carrying multiple such variants. If the multiplicative interaction model is correct, multi-locus association with disease in the artificial population should be detectable and provide insight into the prevalence, magnitude and variance of interaction effects giving rise to highly penetrant disease phenotypes.

## Materials and Methods

### *Drosophila* Stocks and Crossing

*yw*, *w^1118^*, *w;Sco/Cyo;TM3/TM6B* stocks were obtained from the *Drosophila* Stock Center (Bloomington, IN; http://flystocks.bio.indiana.edu). Highly Inbred Line (HIL), *WE70 Drosophila melanogaster* was originally derived from a peach orchard in West End, North Carolina [26]. F_1_ flies were produced from the cross between *yw* males and *WE70* virgin females. F_2_ were generated by random mating of F_1_ male and virgin females. The two 2^nd^ and 3^rd^ chromosome replacement lines *WE70/WE70;+/+* and *+/+;WE70/WE70*, resp., are produced from three generation crosses between *w^1118^* and *WE70* using double balancer lines. The Recombinant Inbred Lines (RILs) were produced from inbreeding crosses between *WE70* and *yw*, which were expected to be nearly homozygous at all loci. At the F_1_ generation, 200 individual pairs were selected, and their progeny were inbred by full-sib mating for more than 10 generations. 152 lines that survived inbreeding were maintained by small mass mating of ∼50 pairs. All flies were maintained under room temperature (21–22°C) on normal food source made from a combination of yeast, corn starch, and molasses [36]. Only female flies at 7 days of age were analyzed using Semi-automated Optical Heart Assays (SOHA) [31] to monitor the heart phenotypes.

### Semi-automated Optical Heart Analysis (SOHA)

SOHA was performed as outlined in Ocorr et al. and Fink et al. [27], [37]. Artificial hemolymph with 10 mM sucrose and 5 mM trehalose were used as semi-dissection solution (100 ml hemolymph, 500 µl sucrose and 250 µl trehalose). Hemolymph solution pH is set at 7.1. Before manipulation the solution was heated for 2 seconds by microwave and saturated with pressurized air for 20 minutes. 1-week old female flies were anaesthetized with five minutes of FlyNap before semi-dissection. Under a microscope, the head, thorax and internal organs of the abdomen were removed from a fly immobilized in a gel, and these partial dissections were maintained in artificial hemolymph solution for 20 minutes prior to video imagining. All procedures were performed at room temperature (21–22°C).

Image analysis: 30 sec movies were obtained for each semi-dissected fly using a high-speed Hamamatsu EMCCD 9300 camera under 10× lens in artificial hemolymph solution. The rate of film was 100–130 frames per second. Around 5000 frames were generated for each 30 sec movie. Custom MatLab software (Mathworks, Natick MA) was used to analyze 30 second of each in order to generate parameters associated with heart function in detail, including M-mode analysis [25], [29]. Arrhythmia index (AI), diastolic diameter (DD) and systolic diameter (SD) of each fly were recorded from the F_2_ generation has a unique heart phenotypic profile from the movie analysis. Each phenotype is linked to its corresponding collected tissue, which was used to generate genomic pools for the SFP-BSA analysis according to the corresponding heart phenotypic parameters.

### Phalloidin Staining

Phalloidin staining was performed as described in Mery et al. and Cammarato et al. [24], [25], [29], [31]. Before fixation each cardiac tube was examined to ensure contractions were inhibited in relaxing buffer containing 10 mM EGTA.

### Single Feature Polymorphism (SFP) and Bulked Segregant Analysis Mapping (BSA)

Four genomic pools with three biological replicates each were generated: two of them from the *yw* and WE70 parental lines that were used to call SFPs between the two parents, another two from F_2_ individuals with extreme aberrant systolic diameter and arrhythmia phenotypes measures from the SOHA heart phenotypic profiles. For each biological replicate genomic DNA was generated from 15 parental or selected F_2_ flies using DNA Easy prep kit (Qiagen). To avoid RNA contamination, genomic DNA was treated with 1.5 µl RNAse at 37°C for 30 min. Linear amplification of genomic DNA was performed using the REPLI-g midi kit to a concentration of ∼ 1 µg/µl. Five µg of amplified genomic DNA was fragmented with 1 U RQ1 DNase (Promega) for 2 min at room temperature (18–22°C) in standard buffer followed by incubation with 2 ul EDTA at 65°C for 10 min, and digestion was confirmed on 3% agarose gels by the existence of sheared products with ∼50–100 bp length. 50 µl fragmented genomic DNA was used in the labeling reaction with 22.5 U terminal deoxynucleotidyl transferase (rTDT) (Promega) and 1 µL (1 mM) Biotin N6-ddATP (Enzo) under the following temperature cycle: 37°C for 90 min, 99°C for 15 min and 12°C for 5 min. Gel-shift assays were performed to confirm labeling efficiency. Subsequently, the labeled products were hybridized to Affymetrix Chips (*Drosophila* Genome 2.0) using standard Affymetrix protocols at the Salk Institute’s Genomic Facility Center. The SFP dataset with raw files have been deposited in NCBI’s Gene Expression Omnibus and are accessible through GEO Series accession number GSE45123 (http://www.ncbi.nlm.nih.gov/geo/query/acc.cgi?acc=GSE45123).

### Statistical Methods

All statistical analysis code was written in R language [38]. The required coordinate file for SFP analysis of Drosophila Genome 2.0 was obtained from Bioconductor. Briefly, the raw.CEL files from scanned arrays were read into R as the log-transferred intensity of 25 mer perfect match (PM) probes after background correction and quantile normalization to ensure that all arrays have the same overall distribution. As an initial quality check, we confirmed that the correlation among replicates was consistently greater than to arrays of different genotypes. Furthermore, all 12 arrays were evaluated with >99% present call using MAS5 algorithm assuming high quality of hybridization. SFP calling was performed using two parental CEL files with 3 replicates each (3 WE70 arrays vs. 3 *yw* arrays), using SAM [39]. In brief, the “relative difference” 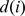
in PM probe intensity is:
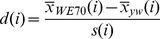
(1)where 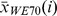
and 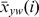 are defined as the average intensity of PM probe 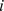 in WE70 and *yw*, respectively. The 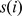 is the standard deviation of repeated intensity of PM probe i:
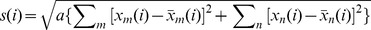
(2)where 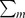
and 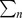 are summations of square of the intensity difference of PM probe 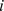 in WE70 and yw, respectively, 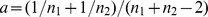, 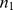 and 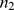 are the numbers of replicates in WE70 and yw. 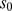 is defined as the median standard deviation of log feature intensity. 20 possible permutation tests were performed according to SAM. The expected null distribution of d-statistics was defined as the average PM probe intensity from each permutation. The FDR (False Discover Rates) was defined as:
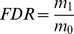
(3)where 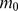 is the average number of features threshold in 20 permutations, 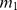 is the number of features above the threshold in the non-permuted data. The absolute WE70 derived allele frequency in each aberrant genomic pool was estimated by
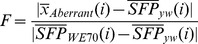
(4)where 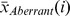 is the average intensity of 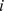
th SFP in either aberrant systolic or arrhythmia genomic pool, 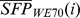 and 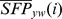 are the average intensity of 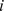
th SFP in two parent genomic pools. The relative WE70 derived allele frequency between two aberrant genomic pools was derived from the difference of two absolute allele frequencies from above [26].

### RIL Sequencing

Six RILs indicated in Figure 5 were chosen at either extreme for both traits, and genomic DNA was sequenced to an average 8X coverage on a HiSeq2000 at the Emory University Cancer Center Genome Sequencing Core. Reads were aligned to the *Drosophila* reference genome and 146,737 SNP variants (Table S4) were extracted from each consensus alignment. Genotypes were called relative to the reference as homozygous variant, or homozygous reference, according to the most common allele in the pile-up, recognizing that only approximately two thirds of the sites are fixed in each line. We then filtered the calls to ensure that each genotype was present in at least 3 of the RILs. For each site, we identified the more common allele across the 23 RIL for which data were available (sequencing of one of the narrow SD RIL failed), and computed the difference between the number of this allele in the six high and six low arrhythmia lines (or six wide and five narrow SD lines). These differences were then averaged over sliding windows of 100 consecutive SNPs, generating values between +6 and −6, where these extremes would correspond to the expectation for a fully penetrant causal variant. Permutations of random sets of RIL drawn from all four phenotypes suggest an approximate threshold of plus or minus 1.8 for observing random peaks covering at least 10 MB. There are several regions that exceed this threshold, notably a major locus on 3R for SD as well as minor peaks on 2L and 2R, and centromere-proximal regions on chromosome 2 for AR.

## Supporting Information

Figure S1 Correlation between the arrhythmia index (AI) and the systolic diameter (SD) phenotypes in all 170 RILs. Pearson correlation between AI and SD was non-significant (R = 0.07, t = 0.86, p = 0.39).
(TIFF)

Figure S2 Correlation between the diastolic diameter (DD) and the systolic diameter (SD) phenotypes in all 170 RILs. Pearson correlation between AI and DD was non-significant (R = 0.94, t = 35.21, p<0.0001).
(TIFF)

Figure S3 Distribution of phenotypes in RIL. Diastolic diameters (DD) of RIL are ranked from smallest to biggest.
(TIFF)

Table S1 Heart phenotype of *yw*, WE70, F_1_ and F_2_ is generated from individual fly movie analysis, which is recorded by Semi-automated Optical Heart Assays (SOHA).
(XLSX)

Table S2 Raw data for SFP analysis with meta-data information of Affymetrix *Drosophila* 2.0 chips. This file includes the raw probe intensities for each of three Affymetrix SFP replicate arrays. Only SFP-identified probes between *yw* and WE70 are indicated. The intensities of probes then are used to calculate the frequency differences between genotype (see main manuscript).
(XLSX)

Table S3 Heart phenotype assessment of 170 RILs generated from fly heart movies and analysis, recorded by Semi-automated Optical Heart Assays (SOHA) [25], [31]. The heart phenotype parameters of each RIL is calculated from 6 to 12 individual movies.
(XLSX)

Table S4 Gene list detected in 5 or more RIL lines with stop codons according to flybase. Of the 147 stop codons, 53 of the associated genes have unknown functions and 37 have functions related to metabolism.
(XLSX)

Video S1 Normal heart beat movie. A 30 sec MP4 video file of a dissected wildtype *yw* fly heart showing a normally beating heart at 1-week of age.
(AVI)

Video S2 Abnormal heart beat movie. A 30 sec MP4 video file of a dissected WE70 fly heart showing an abnormally beating heart at 1-week of age.
(AVI)
