# Supplementary figures and images for: Complex Genetic Architecture of Cardiac Disease in a Wild Type Inbred Strain of Drosophila melanogaster
Source: PLoS One. 2013 Apr 29;8(4):e62909. doi: 10.1371/journal.pone.0062909 (PMC3639251; doi:10.1371/journal.pone.0062909)

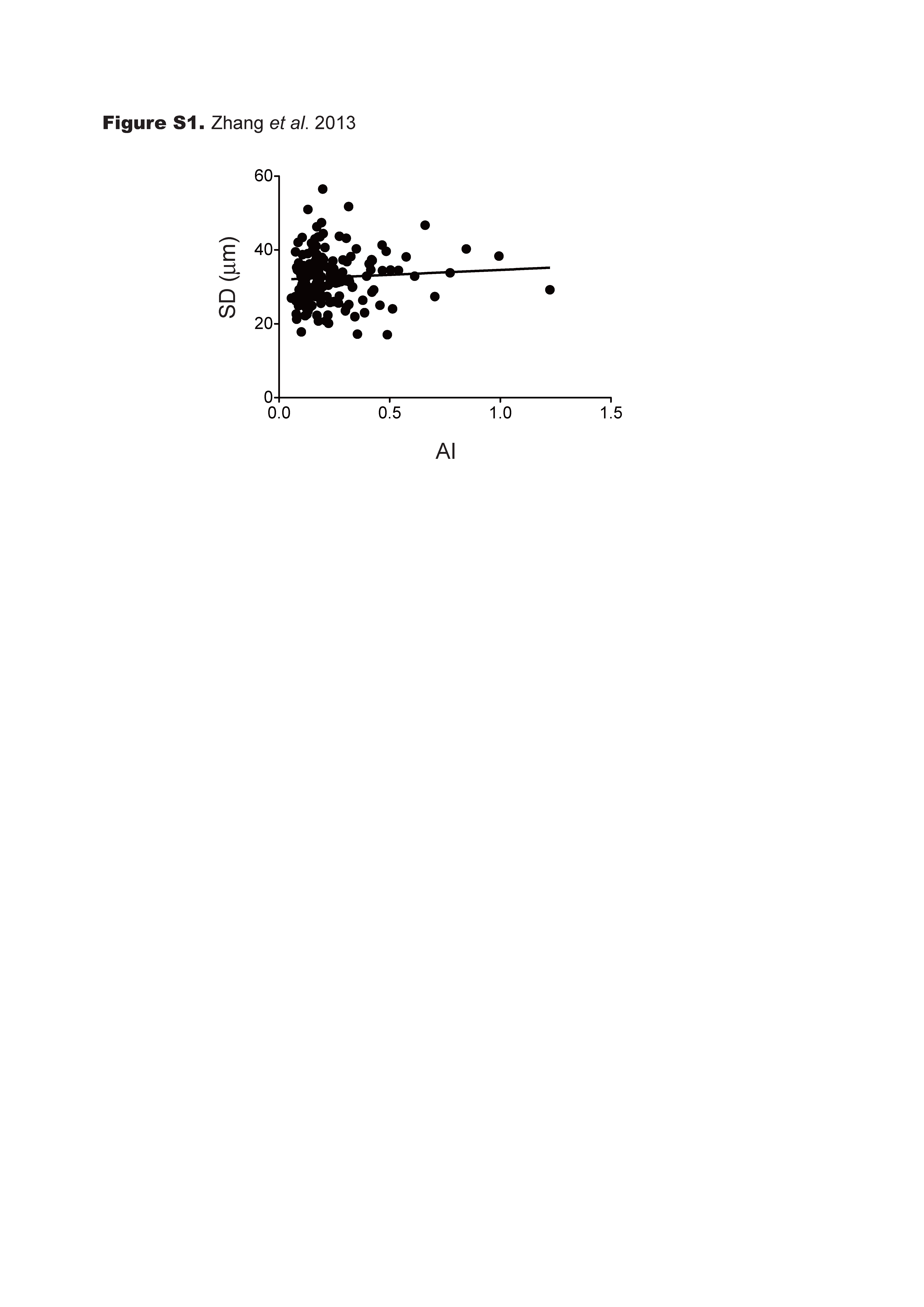

Supplement: Figure S1 — Correlation between the arrhythmia index (AI) and the systolic diameter (SD) phenotypes in all 170 RILs. Pearson correlation between AI and SD was non-significant (R = 0.07, t = 0.86, p = 0.39). (TIFF) [file pone.0062909.s001.tiff]
